# Diagnostic delay and histopathological subtyping challenges in idiopathic multicentric Castleman disease, not otherwise specified: a case report of a three-hospital odyssey

**DOI:** 10.3389/fmed.2026.1829540

**Published:** 2026-07-20

**Authors:** Fen Zhang, Nan Wang, Ying-yu Wang, Ye Peng, Yan-chen Bao, Hua-zheng Shi, Qian-wen Xiao, Xiao-li Zhang, Xiao-xi Wang, Chun-mei Liao, Jing Pan, Wei-qiang Teng

**Affiliations:** 1 Department of Otorhinolaryngology Head and Neck Surgery, Shanghai 411 Hospital, Shanghai, China; 2 Department of Radiology and Nuclear Medicine, Changhai Hospital, Shanghai, China; 3 Shanghai University Affiliated Universal Medicine Imaging Diagnostic Center, Shanghai, China; 4 Department of Pathology, Shanghai 411 Hospital, Shanghai, China; 5 Ouyang Community Health Center of Hongkou District, Shanghai, China

**Keywords:** Castleman disease, fever, hemopathy, iMCD-NOS, lymphadenopathy, tumor

## Abstract

**Background:**

Castleman disease (CD) is a group of rare and heterogeneous lymphoproliferative disorders. Accurate diagnosis and subtyping remain challenging. This report describes a rare case of idiopathic multicentric Castleman disease, not otherwise specified (iMCD-NOS), highlighting the causes of misdiagnosis, diagnostic strategy, and critical features of clinical subtyping.

**Case presentation:**

A 53-year-old male presented with a 4-month history of persistent right submandibular lymphadenopathy. During this period, he experienced two febrile episodes (max temp 39.0 °C), each lasting 2 days, without associated respiratory symptoms. The fever resolved following intravenous antibiotic therapy at a local hospital, however, the lymphadenopathy did not improve and progressively enlarged. Core needle biopsy of the right submandibular lymph node at another municipal hospital revealed acute and chronic inflammation with fibrosis. He also reported occasional low-grade fever and night sweats, prompting admission to our hospital for further evaluation. On admission, physical examination revealed the largest lymph node measuring approximately 2.5 × 2.0 cm and purplish-red papules on the bilateral lower extremities. Laboratory findings revealed leukocytosis, eosinophilia, platelet (PLT) count: 296 × 10^9^/L (Normal), elevated C-reactive protein (66.31 mg/L), and increased interleukin-6 (13.78 pg./mL). PET-CT demonstrated metabolically active lymphadenopathy involving the bilateral cervical, submandibular, supraclavicular, retroperitoneal, and pelvic regions, suggestive of a neoplastic process. Under general anesthesia, an excisional biopsy of a right cervical lymph node was performed. Histopathological examination revealed disrupted lymph node architecture, with the presence of both atrophic and hyperplastic follicles. A subset of follicles showed an “onion-skin” appearance, accompanied by hyaline degeneration of small vessel walls in the paracortical region. The borders between the paracortex and lymphatic sinuses were indistinct, and proliferation of lymphocytes and plasma cells was observed in the sinus areas. Immunohistochemistry demonstrated CD38 positivity in the cortex and medullary sinuses, CD138 positivity in the cortex and paracortex, as well as positive staining for IgG4 and IgG. The pathological diagnosis was mixed-type Castleman disease. In the light of the patient’s multicentric lymph node involvement, systemic inflammatory symptoms, elevated inflammatory markers, and the exclusion of HHV-8 infection as well as features suggestive of TAFRO or IPL, a final diagnosis of iMCD-NOS was made. Given the rarity of the disease and the limited experience with its management in our department, the patient was referred to the Department of Hematology at Ruijin Hospital, Shanghai Jiao Tong University School of Medicine. Following treatment with siltuximab plus prednisone, the patient’s symptoms improved markedly, with resolution of fever and no recurrence of submandibular or cervical lymphadenopathy.

**Conclusion:**

This case illustrates the diagnostic challenges of idiopathic multicentric Castleman disease-not otherwise specified (iMCD-NOS) and underscores the importance of excisional biopsy over core needle biopsy, systematic exclusion of differential diagnoses, and multidisciplinary collaboration. The patient’s journey through three hospitals before diagnosis offers three key lessons for clinicians: (1) The possibility of Castleman disease should be considered when a triad of unexplained lymphadenopathy, systemic symptoms, and elevated inflammatory markers is present. (2) When multicentric lymphadenopathy coexists with systemic inflammation, excisional biopsy—rather than core needle biopsy—is essential for an accurate diagnosis. (3) Systematic exclusion of HHV-8 infection, TAFRO/IPL features, and other mimickers is mandatory for proper iMCD-NOS subtyping. Enhanced awareness of this disease, as provided by this report, may facilitate early recognition and reduce diagnostic delays.

## 1 Introduction

Castleman disease (CD) is a group of rare lymphoproliferative disorders characterized by distinctive histopathological features. Histopathologic examination of the lymph node remains the gold standard for diagnosis (1–3). Clinically, CD is divided into unicentric Castleman disease (UCD) and multicentric Castleman disease (MCD) forms based on the anatomic distribution of involved lymph nodes (2, 4). Additionally, in 2025, researchers including Pierson identified a new clinical subtype, which was classified as Type 3 and named OligoCD (5). UCD involves a single lymph node region, whereas MCD is characterized by multicentric lymphadenopathy and multiorgan involvement, often accompanied by constitutional symptoms (6). OligoCD is characterized by lymph node involvement in ≥2 anatomical regions (without systemic involvement as seen in iMCD) and does not meet the diagnostic criteria for iMCD (5). Among these, iMCD-not otherwise specified (iMCD-NOS) remains a diagnostic challenge due to its nonspecific clinical manifestations, overlapping features with other systemic inflammatory and neoplastic conditions, and the absence of a definitive biomarker.

Diagnosing iMCD-NOS is particularly challenging, as it is frequently initially mistaken for more common conditions, such as infectious lymphadenitis, autoimmune diseases, or lymphoma. Misdiagnosis may lead to unnecessary procedures, delayed appropriate treatment, and disease progression. Moreover, accurate subtyping is critical because treatment strategies and prognoses differ substantially between UCD, HHV-8-associated MCD, and iMCD subtypes (including TAFRO and IPL variants).

Herein, we report a case of iMCD-NOS in a 53-year-old male whose initial presentation included persistent submandibular lymphadenopathy and recurrent fever, leading to a diagnostic odyssey across three hospitals. This case highlights the common pitfalls in diagnosing iMCD-NOS, particularly the risk of misdiagnosis when core needle biopsy is used instead of excisional biopsy, and the importance of systematically excluding HHV-8 infection, TAFRO/IPL features, and other mimickers. By analyzing the causes of misdiagnosis and the key points of clinical subtyping, we aim to provide practical lessons to facilitate early recognition and reduce diagnostic delays in this rare disease.

## 2 Case presentation

A 53-year-old male presented with a 4-month history of persistent right submandibular lymphadenopathy. During this period, he experienced two febrile episodes (max temp 39.0 °C), each lasting 2 days, without associated respiratory symptoms. Although a 2-day course of intravenous cefuroxime sodium (1.5 g three times daily) at a local hospital resolved the fever, the enlargement of the right submandibular and bilateral cervical lymph nodes did not improve and continued to progress. He also reported occasional low-grade fever and night sweats. Core needle biopsy of the right submandibular lymph node was performed at another municipal hospital to identify the etiology. Pathological examination revealed acute inflammatory cell infiltration with fibrous tissue proliferation, consistent with an inflammatory lesion. The patient underwent only diagnostic examinations at this hospital without receiving any treatment.

To further clarify the diagnosis, the patient visited our hospital’s Respiratory Department outpatient clinic and was admitted for hospitalization. The preliminary diagnoses were pulmonary infection and right submandibular lymphadenopathy.

On admission, physical examination revealed several palpable, non-tender, firm, enlarged lymph nodes with slightly reduced mobility in the right submandibular and bilateral cervical regions; the largest measured approximately 2.5 × 2.0 cm. Additionally, diffuse pale purple papules were noted on the skin of both lower extremities. (Figures 1A–D). The patient had no prior history of trauma or surgery, and no familial predisposition to Castleman disease or related genetic disorders has been documented thus far. Laboratory findings: Inflammatory response: White blood cell (WBC) count: 19.39 × 10^9^/L; absolute eosinophil (EOS) count: 0.81 × 10^9^/L; platelet(PLT) count: 296 × 10^9^/L (Normal); C-reactive protein (CRP) level: 66.31 mg/L; erythrocyte sedimentation rate (ESR): 68 mm/h. Virologic status: EB virus (EBV) nuclear antigen IgG antibody>50 AU/mL; EB virus (EBV) capsid antigen IgG antibody>50 AU/mL; Cytokine profile: interleukin-4 (IL-4 L): 8.29 pg./mL, interleukin-6 (IL-6 L): 13.78 pg./mL; TNF-*α*: 4.84 pg./mL, TNF-*β*: 3.16 pg./mL. Serological tests were negative for HIV, HHV-8 DNA, IgG, IgG4, syphilis, hepatitis B, hepatitis C, tuberculosis, and autoimmune disorders. Contrast-enhanced MRI of the neck: multiple lymphadenopathies (Figures 2A–C). PET-CT demonstrated metabolically active lymphadenopathy involving the bilateral cervical, submandibular, supraclavicular, retroperitoneal, and pelvic regions, suggestive of a neoplastic process. After clinical examinations and otolaryngology consultation, the patient was transferred to our department, where an excisional biopsy of a right cervical lymph node was performed under general anesthesia. Histopathological examination revealed disrupted lymph node architecture, with the presence of both atrophic and hyperplastic follicles. A subset of follicles showed an “onion-skin” appearance, accompanied by hyaline degeneration of small vessel walls in the paracortical region. The borders between the paracortex and lymphatic sinuses were indistinct, and proliferation of lymphocytes and plasma cells was observed in the sinus areas (Figures 3A–C). Immunohistochemistry demonstrated CD38 positivity in the cortex and medullary sinuses, CD138 positivity in the cortex and paracortex, as well as positive staining for IgG4 and IgG. The IgG4/IgG ratio was <40%. Based on these findings, the pathological diagnosis was mixed-type Castleman disease. (Clinical findings and diagnostic Assessment are detailed in Table 1).

**Figure 1:**
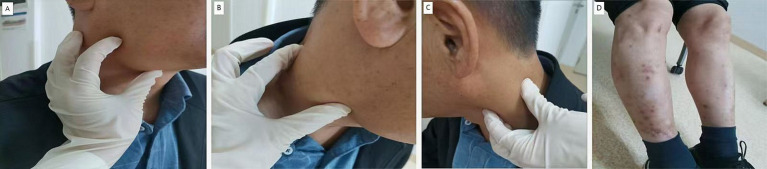
Physical examination findings. **(A)** Enlarged right submandibular lymph node, approximately 2.5 cm × 2.0 cm in size, firm in consistency, with moderate mobility; **(B)** Enlarged right cervical lymph node,approximately 2.0 cm × 2.0 cm in size, firm in consistency, with moderate mobility; **(C)** Enlarged left cervical lymph node, approximately 2.0 cm × 1.5 cm in size, firm inconsistency, with moderate mobility; **(D)** Violaceous papules on bilateral lower extremities.

**Figure 2:**
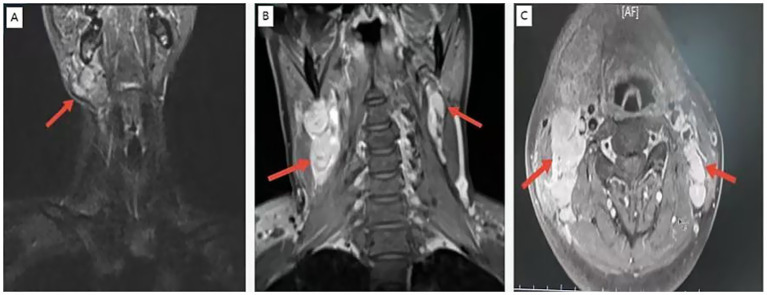
Imaging findings, Contrast-enhanced MRI of the neck. **(A)** Coronal view shows a well-circumscribed enlarged right submandibular lymph node. **(B)** Coronal view reveals multiple enlarged bilateral cervical lymph nodes with marked enhancement. **(C)** Axial view demonstrates multiple enlarged bilateral cervical lymph nodes with restricted diffusion and marked enhancement.

**Figure 3:**
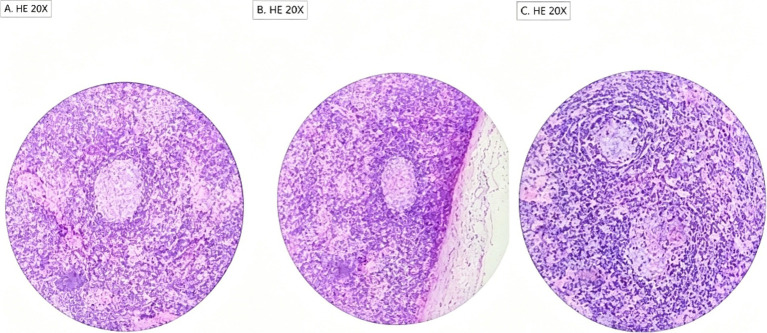
Pathological findings of the right cervical lymph node. **(A, B, C)** Histopathological examination (HE staining, low-power microscope) reveals disrupted lymph node architecture with increased numbers of lymphoid follicles extending into the medullary region. The majority of follicles are atrophic, while a minority show hyperplastic, expanded germinal centers. Occasional follicles exhibit an “onion-skin” appearance. Hyalinization of small blood vessel walls is observed in the paracortical region. The boundary between the paracortex and lymph node sinuses is indistinct, with evidence of sinusal lymphoplasmacytic infiltration. Focal aggregates of neutrophils forming “microabscesses” are present.

**Table 1:** Clinical findings and diagnostic assessment.

Purpose	Project	Find results
Inflammatory response	Complete blood count (CBC), C-reactive protein (CRP), erythrocyte sedimentation rate (ESR), liver and kidney function tests, urinalysis, lymphocyte subpopulations, sputum bacterial culture	White blood cell (WBC) count: 19.39 × 10^9^/L; platelet(PLT)count: 296 × 10^9^/L; hemoglobin (Hb): 138 g/L; C-reactive protein (CRP) level: 66.31 mg/L; erythrocyte sedimentation rate (ESR): 68 mm/h; glomerular filtration rate (eGFR): 91.93 mL·min^−1^·(1.73 m^2^)^−1^; urinalysis: negative
Virologic status	hepatitis B, hepatitis C, human immunodeficiency virus antibody, *Treponema pallidum*-specific antibody, EB virus antibody	Human herpesvirus 8 DNA (HHV-8 DNA): Negative; All were negative: HIV, hepatitis B, hepatitis C, syphilis
Cytokine profile	Cytokines (flow cytometry)	interleukin-4(IL-4): 8.29 pg./mL, interleukin-6(IL-6): 13.78 pg./mL, TNF-*α*: 4.84 pg./mL, TNF-*β*: 3.16 pg./mL
Tumor-related	Tumor markers	All are normal
Immunology	T-cell detection for tuberculosis infection,immunoglobulins, IgG4,	T-cell testing for tuberculosis infection: Negative; Immunoglobulin G4 subtype (IgG4): 0.68 g/L; Immunoglobulin G (IgG): 13.3 g/L(serum IgG4/IgG = 5.11%);
Organ function	Electrocardiogram	Sinus heart rate; rotating clockwise
Imaging	Chest CT, contrast-enhanced MRI of the neck, and whole-body positron emission tomography-computed tomography (PET-CT).	Chest CT: bilateral pneumonia, and mildly enlarged lymph nodes in the mediastinum; Cervical MRI: multiple lymphadenopathies; PET-CT: hypermetabolic lymph node lesions were observed bilaterally in the neck, right submandibular region, suprascapular region, bilateral pelvic walls, and retroperitoneal area, with the largest lesion measuring approximately 2.0 cm in short diameter, accompanied by splenomegaly.
Pathology	Microscopic examination, immunohistochemistry	Microscopic examination: disrupted lymph node architecture, a small number of folliculi lymphaticus are present: “onion-skin” appearance. Small vessel walls in the paracortical zone show hyaline degeneration, Immunohistochemistry findings include: immunoglobulin G4 (IgG4, positive), and immunoglobulin G (IgG, positive), the IgG4/IgG ratio was <40%. Based on these findings, the pathological diagnosis was mixed-type Castleman disease.

In the light of the patient’s multicentric lymph node involvement, systemic inflammatory symptoms, elevated inflammatory markers, and the exclusion of HHV-8 infection as well as features suggestive of TAFRO or IPL, a final diagnosis of iMCD-NOS was made. Given the rarity of the disease and the limited experience with its management in our department, the patient was referred to the Department of Hematology at Ruijin Hospital, Shanghai Jiao Tong University School of Medicine. According to the risk stratification criteria established by the International Castleman Disease Collaborative Network (CDCN), this patient was classified as “non-severe” and received intravenous siltuximab at a dose of 11 mg/kg every 3 weeks, along with prednisone at an initial dose of 1 mg·kg^−1^·d^−1^, which was gradually tapered and discontinued after 6 weeks. At follow-up after 6 months of treatment, the patient’s CRP levels decreased by more than 50% (from 66.31 mg/L to 23.76 mg/L), with hemoglobin at 150 g/L, albumin at 42.9 g/L, and eGFR at 91 mL/min/1.73 m^2^. The patient no longer experienced fever, and submandibular and cervical lymphadenopathy had resolved, indicating therapeutic efficacy. The siltuximab administration interval has since been extended to 6 weeks, with regular follow-up monitoring (The timeline diagram illustrating the patient’s clinical course and diagnostic/treatment workflow is detailed in Figure 4).

**Figure 4:**
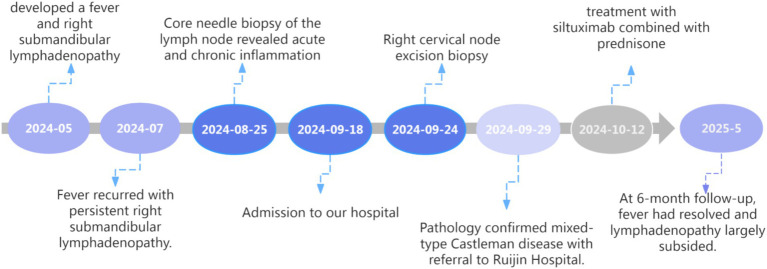
Timeline diagram illustrating the patient’s clinical course and diagnostic/treatment workflow.

## 3 Discussion

Castleman disease (CD) is a group of rare lymphoproliferative disorders, also known as angiofollicular lymph node hyperplasia, giant lymph node hyperplasia, and angiofollicular lymphoid hamartoma. First described in 1954 by the American pathologist Benjamin Castleman (6), it was designated as a rare disease in China’s First List of Rare Diseases by the National Health Commission in 2018. In 2021, the Chinese Castleman Disease Collaborative Network was established to advance research and clinical collaboration (2). The incidence of CD is approximately 1 in 50,000 (7). In the United States, this translates to 6,500–7,700 cases annually, with UCD accounting for 75%. While the overall incidence in Japan is similar, the distribution differs markedly: MCD comprises 70% of cases, and UCD 30% (8). CD most commonly involves the mediastinal and retroperitoneal regions, second most common site is the head and neck (6).

The uniqueness of this case lies in its presentation as a rare subtypes of rare diseases, with the primary challenges being its diagnosis and accurate clinical subtyping. Given the nonspecific clinical manifestations and imaging findings, a common disease was deemed unlikely. This highlights the necessity of a thorough literature review in complex cases to identify subtle diagnostic clues and pursue stepwise, rigorous validation.

1 When presented with painless lymphadenopathy and specific clinical features suggestive of Castleman disease (CD), the diagnostic approach should begin with a thorough exclusion of other disorders that can mimic its histopathologic features. The differential diagnosis was broad, encompassing infectious, neoplastic, and autoimmune etiologies. Infectious causes included HIV, syphilis, tuberculosis, EBV, and toxoplasmosis. Neoplastic considerations comprised lymphoma, POEMS syndrome, and plasmacytoma. Autoimmune diseases such as systemic lupus erythematosus (characterized by an antinuclear antibody [ANA] titer of ≥1:80 on HEp-2 cells) and rheumatoid arthritis should also be considered (2). It is necessary to focus on excluding diseases including lymphoma, IgG4-related disease, Rosai-Dorfman disease, and Kimura disease (The differential diagnosis of these four diseases is detailed in Table 2).

**Table 2:** Differential diagnosis.

Item	Lymphoma (20–24)	IgG4-RD (10, 11)	Rosai-Dorfman disease (12)	Kimura disease (13)	Castleman disease (1)
Patholo-gical Feature-s	Reed-Sternberg (R-S) cells and variant R-S cells; "mirror cells”; "popcon kernel.” Low-grade NHL versus CD is critical: ① A follicular growth pattern composed of centrocytes and centroblasts and carry the t(14;18)(q32;q21) translocation; ② Monoclonal expansion of predominantly small B-lymphocytes with variable plasmacytoid differentiation; ③ This low-grade neoplasm consists mainly of small B-lymphocytes, along with admixed prolymphocytes and paraimmunoblasts. ④ Cells display heterogeneous morphology, including indented nuclei with scant cytoplasm, monocytoid features, or plasmacytic cells	1. Clinical examination showing characteristic diffuse/localized swelling or masses in single or multiple organs 2. Hematological examination shows:elevated serum IgG4 concentrations (≥135 mg/dl) 3. Histopathologic examination shows: (1) Marked lymphocyte and plasmacyte infiltration and fibrosis. (2) Infiltration of IgG4 + plasma cells: ratio of IgG4+/IgG + cells > 40% and >10 IgG4 + plasma cells/HPF. Definite: 1 + 2 + 3 Probable: 1 + 3 Possible: 1 + 2	Thesinus expansion of large histiocytes, aspossessing ample pale or “watery-clear” cytoplasm with a largehypochromatic nucleus and prominent nucleolus. Nodal RDD is often accompanied by numerous plasma cells inthe medullary cords and around the venules, with varying proportions of IgG4/IgG plasma cells. Consistent features, regardless ofthe site, include the cytomorphology of the large pale histiocytesand their immunophenotype. Extranodal lesions are usually associated with morefibrosis, fewer RDD histiocytes and less emperipolesis. The immunophenotype of the large RDD histiocytes is characterized by cytoplasmic and nuclear S100 and fascin positivity, with CD68 and variable CD163 and CD14 positivity.	Follicular hyperplasia Reactive germinal centers Marked eosinophilia Eosinophilic microabscesses Preserved nodal architecture Warthin–Finkeldey polykaryocytes Capsular fibrosis	HV-CD: “onion-skin” appearance; The vascular walls may demonstrate hyaline degeneration, resulting in a “lollipop” -like appearance. PC-CD: Significant increase in plasma cell infiltration, which in some cases can completely replacing the normal structure of this region. In most cases, the plasma cells exhibit mature morphology.

To summarize the key points for differential diagnosis of the above four diseases: Lymphoma, IgG4-related disease, Rosai-Dorfman disease, and Kimura disease lack distinct clinical features that allow for clear differentiation; their differential diagnosis is currently primarily based on pathological examination.

(1) The differential diagnosis of CD encompasses both NHLs and cHL, depending on the morphological subtype. In the HV subtype, the main differential diagnoses include follicular lymphoma and MZL. In the mixed and plasma cell subtypes, the differential diagnosis additionally includes MZL with terminal differentiation, immunocytoma, cHL, and T-cell NHLs, particularly the nTFH-AI type. Comprehensive immunohistochemical analysis combined with clonality studies is generally sufficient to establish the correct diagnosis.
(2) IgG4-related lymphadenopathy, defined as lymphadenopathy developing in patients with IgG4-related sclerosing disease. IgG4-RD manifests with systemic nonspecific symptoms (e.g., fatigue, weakness, weight loss) and organ-specific features, most commonly involving the secretory glands, bile ducts, and kidneys. Characteristic findings include diffuse enlargement or localized swelling of affected organs, often with mass or nodular formation. The disease typically follows a relapsing and progressive course, ultimately resulting in vital organ dysfunction or multi-organ failure. Is known to manifest in 5 histologic patterns: (1) multicentric Castleman disease-like; (2) reactive follicular hyperplasia; (3) interfollicular plasmacytosis with immunoblasts; (4) progressive transformation of germinal centers-like; and (5) inflammatory pseudotumor-like (9). Among these patterns, the “multicentric Castleman disease-like” subtype closely resembles Castleman disease. A definitive diagnosis of IgG4-related diseases requires not only that the pathological criteria listed below be met: Marked lymphocyte and plasmacyte infiltration and fibrosis, Infiltration of IgG4 + plasma cells: ratio of IgG4+/IgG + cells > 40% and >10 IgG4 + plasma cells/HPF, the following two relevant clinical manifestations must also be met: characteristic diffuse/localized swelling or masses in single or multiple organs, hematological examination shows elevated serum IgG4 concentrations (≥135 mg/dL) (10, 11). The patient’s serum IgG4 level was 68 mg/dL, and the ratio of IgG4 to IgG observed in pathological findings was below 40%.
(3) Rosai-Dorfman diseaseclinical clinical manifes-tation Often involves multiple systems: ① Constitutional: Fever, night sweats, fatigue; ② HEENT: Cervical swelling; diplopia; retro-orbital pain; eyelid/lacrimal gland swelling; nasal obstruction; epistaxis; hyposmia; oral ulcers or pain; ③ Cardiovascular: Dyspnea, orthopnea; ④ Pulmonary: Dyspnea, cough; ⑤ Gastrointestinal: Abdominal pain, constipation, hematochezia; ⑥ Renal: Hematuria, flank pain; ⑦ Musculoskeletal: Bone pain; ⑧ Dermatologic: Rash, pruritus; ⑨ Endocrine: Polydipsia/polyuria. Pathological features of Rosai-Dorfman disease (RDD): The diagnostic pathologic features of nodal RDD include thesinus expansion of large histiocytes, aspossessing ample pale or “watery-clear” cytoplasm with a largehypochromatic nucleus and prominent nucleolus. Nodal RDD is often accompanied by numerous plasma cells inthe medullary cords and around the venules, with varying proportions of IgG4/IgG plasma cells. Consistent features, regardless ofthe site, include the cytomorphology of the large pale histiocytesand their immunophenotype. The immunophenotype of the large RDD histiocytes is characterized by cytoplasmic and nuclear S100 and fascin positivity, with CD68 and variable CD163 and CD14 positivity (12). The pathological morphology and immunophenotype of this patient do not align with these findings.
(4) Kimura disease clinically presents as multifocal subcutaneous masses of the head and neck, involving soft tissue, cervical lymph nodes, and salivary glands. Characteristic clinical features include peripheral eosinophilia, eosinophilic infiltrate within the masses, and elevated IgE levels. It is distinguished from other conditions by its characteristic histopathological features, including follicular hyperplasia, reactive germinal centers, marked eosinophilia, eosinophilic microabscesses, preserved nodal architecture, Warthin–Finkeldey polykaryocytes, and capsular fibrosis. Elevated peripheral blood eosinophils and serum IgE levels are also characteristic features of this disease (13). In this patient, both peripheral blood eosinophils and serum IgE levels were elevated. However, the following pathological features were absent: marked eosinophilia, Warthin-Finkeldey polykaryocytes, and capsular fibrosis.

The patient initially presented to three different hospitals. Repeated anti-infective therapy was administered. However, a systematic workup was not performed. This prevented a definitive diagnosis and relevant differential diagnosis.

The second step, imaging and physical examination determined the extent of nodal involvement. Clinical presentation then guided accurate subtyping. Imaging findings in this patient are consistent with Castleman disease, which lacks specific radiographic features. While imaging can aid in distinguishing UCD from MCD, it does not provide definitive evidence for diagnosis.

Ultimately, excisional lymph node biopsy is the gold standard for diagnosing Castleman disease. This patient initially underwent a needle biopsy at a local hospital, which revealed onlyinflammatory changes. This case highlights that needle biopsies carry a risk of missed or incorrect diagnosis. Therefore, excisional biopsy of the entire lymph node remains the more reliable diagnostic approach.

The diagnostic efficacy of needle biopsy and excisional biopsy for CD differs as follows: In a study by Bustamante, among the 110 patients with iMCD, there were 49 females (45%) and 61 males (56%). Thirty-two (29%) patients received a core needle biopsy before an excisional biopsy, while 78 (71%) patients began with an excisional biopsy. The median time from first biopsy to diagnosis was significantly shorter for patients who began with an excisional biopsy (*W* = 1893, *p* = 1.0 × 10^−6^), indicating that core needle biopsies delay diagnosis. From the 110 iMCD patients, only 2 (2%) patients were diagnosed from a core biopsy. Core needle biopsies are rarely sufficient for an iMCD diagnosis and delay an accurate diagnosis (14).

Clinical presentations and subtypes of CD vary by lymph node involvement. 1. UCD involves a single lymph node region. Most patients are asymptomatic aside from localized lymphadenopathy. However, a subset may present with systemic inflammatory symptoms similar to MCD, including fever, night sweats, weight loss, and anemia. UCD can also be associated with paraneoplastic pemphigus or bronchiolitis obliterans (15, 16). 2. MCD is defined by the involvement of multiple (two or more) lymph node stations, with affected nodes having a short-axis diameter of at least 1 cm (1). Unlike UCD, MCD involves more than just lymphadenopathy. Patients frequently present with systemic manifestations, including fever, night sweats, fatigue, and weight loss. Additional common findings are anemia, hepatic insufficiency, and renal insufficiency. Volume overload — such as anasarca, pleural effusions, and ascites—is also a characteristic feature (2). 3. OligoCD is a novel subtype for which no standardized diagnostic criteria currently exist. Its key features are as follows: (1) Lymphadenopathy in ≥2 anatomical regions (but not generalized as in iMCD); (2) Does not fulfill iMCD diagnostic criteria; (3) Not UCD (since UCD involves only a single region); (4) Often presents with a regional pattern of lymph node involvement; (5) Clinical behavior and prognosis are closer to UCD (typically indolent) than to iMCD (more systemic and aggressive) (5). Based on these diagnostic criteria, the patient’s pathology was consistent with CD and involved multiple lymph node regions. The case was therefore classified as MCD.

HHV-8 infection is highly prevalent in MCD and may be associated with its pathogenesis (6). MCD is further classified by HHV-8 status. It is divided into HHV-8-positive and HHV-8-negative MCD (1, 4). HHV-8-negative MCD is further classified into two subtypes: asymptomatic MCD (aMCD) and idiopathic MCD (iMCD). aMCD presents with lymphadenopathy alone, without systemic symptoms or hyperinflammatory features. iMCD is characterized by systemic symptoms and/or evidence of organ dysfunction (2). Therefore, based on the pathological findings and clinical manifestations of this case, the patient was diagnosed with iMCD. (Diagnostic criteria for iMCD and findings in the present case are detailed in Table 3).

**Table 3:** Diagnostic criteria for iMCD and findings in the present case.

Item		International Diagnostic Criteria (2017) (3)	China Diagnostic Criteria (2025) (1)	Findings in the present case
Main diagnostic criteria (both must be met)		Histopathologic lymph node features consistent with the iMCD spectrum Features along the iMCD spectrum include: Regressed/atrophic/atretic germinal centers+ “onion skinning” mantle zone, vascular/FDC proliferation with lollipop-shaped patterns, and patchy increase of plasma cells in the interfollicular zone	Lymph node pathology is consistent with CD: (1) Hyaline vascular subtype of CD (HV-CD): The classic “onion skinning + lollipop”appearance. (2) Plasma cell subtype of CD (PC-CD): “Plasma cell proliferation.” (3) Mixed-type CD (mixed type of CD): exhibits morphological characteristics combining those of both HV-CD and PC-CD	Microscopic examination: Lymph nodes are present with increased number of lymphoid follicles extending into the medullary region; most exhibit atrophic changes, while a minority demonstrate hyperplastic expansion of germinal centers and a few show an “onion-skin” appearance. The walls of small vessels in the paracortical area exhibit hyaline degeneration, and lymphocytes and plasma cells proliferate in the sinusoidal areas
		2. Enlarged lymph nodes (≥1 cm in short-axis diameter) in ≥2 lymph node stations	2. Involvement of ≥2 lymph node regions (short diameter of lymph nodes ≥1 cm)	2. ① Chest CT: Mildly enlarged lymph nodes within the mediastinum; ② Thyroid and cervical lymph node ultrasound and Cervical MRI: Multiple lymph node enlargementin the neck; ③ PET-CT: Hypermetabolic lymph node lesions were observed bilaterally in the neck, right submandibular region, suprascapular area, bilateral pelvic walls, and retroperitoneal region, with the largest lesion measuring approximately 2.0 cm in short diameter
Secondary diagnostic criteria (must meet at least 2 of the 11 criteria and include at least one laboratory test criterion)	Laboratory diagnosis	Elevated CRP or ESR Anemia Thrombocytopenia Hypoalbuminemia Renal dysfunction Polyclonal hypergammaglobulinemia	Elevated C-reactive protein (CRP)or erythrocyte sedimentation Anemia Thrombocytopenia or thrombocytosis; Serum albumin <35 g/L; Estimated glomerular filtration rate (eGFR) < 60 mL·min^−1^·(1.73 m^2^)^−1^ or proteinuria; Blood serum IgG > 17 g/L	C-reactive protein (CRP) level was 66.31 mg/L (>10 mg/L); erythrocyte sedimentation rate (ESR): 68 mm/h (>15 mm/h);
	Clinical diagno-sis	Constitutional symptoms: night sweats, fever (>38 °C), weight loss, or fatigue Large spleen and/or liver Fluid accumulation: edema, anasarca, ascites, or pleural effusion Eruptive cherry hemangiomatosis or violaceous papules Lymphocytic interstitial pneumonitis	Systemic symptoms: night sweats, fever (body temperature>38 °C), weight loss (≥10% decrease within 6 months), or fatigue; Hepatomegaly and/or splenomegaly; Edema or serous cavity effusion; Cherry angiomas or violet-like papules on the skin; Lymphocytic interstitial pneumonia	During the course of the disease, the patient experienced night sweats, fatigue, and recurrent fever, with a maximum body temperature of 39.0 °C PET-CT examination revealed splenomegaly Diffuse purplish-red maculopapular rash on both lower limbs Chest CT findings: bilateral pneumonia with focal atelectasis in the right lower lobe of the lung

iMCD can be further classified into three subtypes:1. iMCD with thrombocytopenia (T), anasarca (A), fever (F), reticulin fibrosis of the bone marrow (R), and organomegaly (O) (iMCD-TAFRO); 2. Idiopathicplasmacytic lymphadenopathy (iMCD-IPL); 3. Idiopathic multicentric Castleman disease, not otherwise specified (iMCD-NOS) (1, 17).

The diagnostic and exclusion criteria for the clinical subtypes of this patient as follows. iMCD patients with shared manifestations, including thrombocytopenia, anasarca/ascites, reticulin fibrosis in bone marrow, renal dysfunction, organomegaly (TAFRO), and typically normal immuno globulin levels. iMCD patients without TAFRO syndrome typically have thrombocytosis, hypergammaglobulinemia, and less severe fluid accumulation. This non-TAFRO group has been called idiopathic plasmacytic lymphadenopathy with polyclonal hyperimmunoglobuli nemia or IPL-type (3). The patient met only the criteria for fever and lymphadenopathy, lacking the features of thrombocytopenia, anasarca/ascites, bone marrow reticulin fibrosis, renal dysfunction, organomegaly (excluding TAFRO subtype), as well as thrombocytosis, hypergammaglobulinemia, and fluid accumulation (excluding IPL subtype). Therefore, the final clinical subtype of this patient was diagnosed as iMCD-NOS.

Multiple factors have been identified as contributing to this condition, including viral infections (e.g., HIV, HHV-8, EBV), genetic abnormalities, autoimmune processes, and dysregulation of cytokine levels (such as elevated IL-6 concentrations) (18). The pathogenic role of HHV-8 has been well established, although its exact mechanisms remain unclear. Previous studies have demonstrated that excessive secretion and dysfunction of cytokines—including IL-6, IL-10, TNF-*α*, and IL-1—play a pivotal role, with IL-6 serving as the central regulatory factor (19). Based on the clinical manifestations in this case—elevated levels of cytokines such as IL-6 and tumor necrosis factor, as well as positive EBV antibody testing—these findings warrant further investigation into their association with the pathogenesis.

The limitations of this study is as follows: The patient exhibited significant eosinophilia, suggesting that in addition to differentiation from Kimura disease, factors such as parasitic infections (e.g., Toxoplasma gondii) should be considered; however, no parasitic testing was conducted in this case. These limitations compromise the completeness and reliability of the clinical data, warranting further improvement in future research.

In summary, the scientific value of this report lies primarily in the following aspects: (1) emphasizing the importance of early systemic evaluation in patients presenting with nonspecific constitutional symptoms and lymphadenopathy; (2) highlighting the critical role of complete lymph node excision and histopathological examination compared with core needle biopsy, which may be misleading; (3) discussing the differential diagnosis between iMCD and IgG4-related disease, particularly in cases with abundant IgG- and IgG4-positive plasma cells; (4) stressing the importance of an integrated clinicopathological approach for establishing the correct diagnosis.

## Patient perspective

The patient appreciated the clear communication and prompt care, Willing to cooperate with examinations and treatment, and relatively satisfied with the therapeutic outcomes, the patient initiated and enjoyed a new life after discharge.

## Data Availability

The original contributions presented in the study are included in the article/supplementary material, further inquiries can be directed to the corresponding author/s.
